# *Schistosoma mansoni* Sirtuins: Characterization and Potential as Chemotherapeutic Targets

**DOI:** 10.1371/journal.pntd.0002428

**Published:** 2013-09-12

**Authors:** Julien Lancelot, Stéphanie Caby, Florence Dubois-Abdesselem, Mathieu Vanderstraete, Jacques Trolet, Guilherme Oliveira, Franz Bracher, Manfred Jung, Raymond J. Pierce

**Affiliations:** 1 Center for Infection and Immunity of Lille (CIIL), INSERM U1019 – CNRS UMR 8204, Université Lille Nord de France, Institut Pasteur de Lille, Lille, France; 2 Genomics and Computational Biology Group, Center for Excellence in Bioinformatics, National Institute of Science and Technology in Tropical Diseases, Centro de Pesquisas René Rachou, Fundação Oswaldo Cruz, Belo Horizonte, Minas Gerais, Brazil; 3 Department für Pharmazie, Zentrum für Pharmaforschung, Ludwig-Maximilians-Universität, München, Germany; 4 Institut für Pharmazeutische Wissenschaften, Albert-Ludwigs-Universität Freiburg, Freiburg, Germany; Rush University Medical Center, United States of America

## Abstract

**Background:**

The chemotherapy of schistosomiasis currently depends on the use of a single drug, praziquantel. In order to develop novel chemotherapeutic agents we are investigating enzymes involved in the epigenetic modification of chromatin. Sirtuins are NAD+ dependent lysine deacetylases that are involved in a wide variety of cellular processes including histone deacetylation, and have been demonstrated to be therapeutic targets in various pathologies, including cancer.

**Methodology, Principal Findings:**

In order to determine whether *Schistosoma mansoni* sirtuins are potential therapeutic targets we first identified and characterized their protein sequences. Five sirtuins (SmSirt) are encoded in the *S. mansoni* genome and phylogenetic analysis showed that they are orthologues of mammalian Sirt1, Sirt2, Sirt5, Sirt6 and Sirt7. Both SmSirt1 and SmSirt7 have large insertion in the catalytic domain compared to their mammalian orthologues. SmSirt5 is the only mitochondrial sirtuin encoded in the parasite genome (orthologues of Sirt3 and Sirt4 are absent) and transcripts corresponding to at least five splicing isoforms were identified. All five sirtuins are expressed throughout the parasite life-cycle, but with distinct patterns of expression. Sirtuin inhibitors were used to treat both schistosomula and adult worms maintained in culture. Three inhibitors in particular, Sirtinol, Salermide and MS3 induced apoptosis and death of schistosomula, the separation of adult worm pairs, and a reduction in egg laying. Moreover, Salermide treatment led to a marked disruption of the morphology of ovaries and testes. Transcriptional knockdown of *SmSirt1* by RNA interference in adult worms led to morphological changes in the ovaries characterized by a marked increase in mature oocytes, reiterating the effects of sirtuin inhibitors and suggesting that SmSirt1 is their principal target.

**Conclusion, Significance:**

Our data demonstrate the potential of schistosome sirtuins as therapeutic targets and validate screening for selective sirtuin inhibitors as a strategy for developing new drugs against schistosomiasis.

## Author Summary

Schistosomiasis is a disease affecting more than 200 million people in tropical and sub-tropical countries caused by parasitic flatworms of the genus *Schistosoma*. The current reliance on a single drug, Praziquantel, for the treatment and control of the disease renders urgent the development of new therapeutic agents. The strategy that we have chosen is to target the enzymes that carry out epigenetic modifications of the chromatin in the parasite and in particular the histone deacetylases (HDACs). Inhibitors of HDACs have been developed as drugs against cancer and our aim is to exploit structural differences in the catalytic domains of the schistosome enzymes in order to develop selective inhibitors that will be drug precursors. Sirtuins are histone deacetylases that have an NAD+-dependent catalytic mechanism. In this study we have characterized all the *Schistosoma mansoni* sirtuins and show that they are expressed throughout the parasite life-cycle. Sirtuin inhibitors cause the death of schistosome larvae, the separation of adult worm pairs and tissue damage to the worm reproductive organs. These results demonstrate the validity of *S. mansoni* sirtuins as therapeutic targets.

## Introduction

The current strategy for the treatment and control of schistosomiasis is the mass-treatment of populations in endemic areas using the only available drug, Praziquantel. Notably, the Schistosomiasis Control Initiative [1] in sub-Saharan Africa had dispensed more than 40 million doses of Praziquantel by 2008. Although this ongoing programme will undoubtedly have a major impact on morbidity and mortality in the region (estimated at 280000 deaths annually prior to the initiative [2]), this approach renders probable the eventual selection of resistant strains of schistosomes [3], which have already been characterized in endemic areas [4] and can be selected in the laboratory [5]. The development of new drugs is therefore indispensable in order to ensure our capacity to treat schistosomiasis in the long term.

In the search for new drug leads one of the possible approaches is to exploit strategies that have been successful for other pathologies. We have chosen to target a group of enzymes that is under active study for the development of anti-cancer drugs, the enzymes that effect posttranslational modifications of histones including the (de)acetylation and (de)methylation of lysine or arginine residues. Inhibitors of these enzymes have been shown to be generally more toxic for cancer cells than for normal cells [6]. Two such drugs (Vorinostat and Romidepsin), both histone deacetylase (HDAC) inhibitors, have been approved for use in humans and a further 15 HDAC inhibitors are in clinical trials [7].

Our working paradigm is that schistosomes, like other parasites, have some of the characteristics of malignant tumours [8]. Their cell division (for egg production) is intense and outside the control of the host, they are practically invisible to the host immune response. They also have a high level of metabolic activity, which like tumours, is dependent on the use of large amounts of glucose that is metabolized by aerobic glycolysis (culminating with the conversion of pyruvate to lactate rather than its use in oxidative phosphorylation) within the mammalian host [9], [10]. This type of metabolism was first shown to be a characteristic of cancer cells by Warburg [11]. Moreover, the reverse paradigm, that tumour cells behave like parasitic organisms to favour their survival and growth, has also been proposed [12]. This is based on the observation that metabolites (including fatty acids, ketones, glutamine and glucose) from “host” tissues promote tumour growth. The metabolic switch to lactate production in cancer cells has been linked to changes in their epigenetic state [13]. In consequence our expectation is that inhibitors of histone modifying enzymes will be significantly more toxic to the parasite than to the host, and, moreover, that analogues, or novel inhibitors, can be identified that will be selective for the schistosome target. The availability of the annotated genome sequence for *Schistosoma mansoni* [14] has allowed us to identify the schistosome histone modifying enzymes [8]. Here we have studied the *S. mansoni* sirtuins and attempted to evaluate their potential as therapeutic targets.

Sirtuins are NAD^+^-dependent deacetylases that are also referred to as class III HDACs, although they are phylogenetically unrelated to the Zn^2+^-dependent class I and II HDACs [15]. Sirtuins can also act as mono-ADP-ribosyltransferases. For example, human sirtuin 4 (Sirt4) is a mitochondrial enzyme that down-regulates glutamine dehydrogenase by ADP-ribosylation [16]. Moreover, Sirt5 has been recently demonstrated to preferentially hydrolyze succinyl and malonyl lysine [17], whilst Sirt6 has low deacetylase activity, but efficiently removes long-chain fatty acyl groups, such as myristoyl, from lysine residues [18]. Sirtuins can be divided into five classes, one of which (class U) is only represented in bacteria and archaea [19]. The seven human sirtuins are grouped into the four other classes and have distinct subcellular localizations. Sirtuins 3, 4 and 5 are localized in the mitochondria, Sirts 6 and 7 are exclusively nuclear, Sirt1 has a dual nuclear/cytosolic localisation and Sirt2 is cytosolic [20]. Many different target proteins have been described for these enzymes and even the nuclear sirtuins act on proteins other than histones. For instance, Sirt1 deacetylates transcription factors such as p53 [21] and FoxO [22]. In keeping with this variety of substrates, in metazoans sirtuins have been associated with a wide variety of processes including transcriptional silencing, ageing, metabolic regulation and apoptosis [23 for review].

Despite the identification of inhibitors of Sirt1, Sirt2, and Sirt3 with a wide range of core structures [24 for review], the need for potent and selective inhibitors, particularly of Sirt1, remains to be fulfilled [7]. One compound (Selisistat or SEN196) is in Phase II clinical trials for Huntington's disease [7]. However, the demonstration of the activity of sirtuin inhibitors such as Sirtinol, which induces apoptosis and autophagic cell death in MCF-7 human breast cancer cells [25], or Salermide, which targets both Sirt1 and Sirt2 and, in so doing, induces cell death and p53 acetylation, again in MCF-7 cells [26], shows the potential of sirtuin inhibitors in cancer therapy. The inhibition of sirtuins has been less well studied for therapeutic potential against parasites than has the inhibition of class I and II HDACs. In the case of *Plasmodium falciparum* this is partly due to the fact that both of the sirtuins present, PfSir2A and PfSir2B, can be genetically disrupted without an impact on parasite viability *in vitro* [27]. However, both Nicotinamide and the synthetic inhibitor Surfactin inhibit PfSir2 activity and are potent inhibitors of intra-erythrocytic growth of the parasite [28], [29]. Disruption of the gene encoding a cytosolic Sir2 homologue in *Leishmania infantum* showed its necessity for parasite survival and the sirtuin inhibitor Sirtinol inhibited the *in vitro* growth of the parasite via the induction of apoptosis [30].

In the present study we have identified and characterized the five sirtuins encoded in the *S. mansoni* genome and established their homology relationships through phylogenetic analysis. We further investigated the relative expression of their transcripts at different life-cycle stages. We next showed that inhibitors of human sirtuins, including Sirtinol and Salermide, induced the death of schistosomula in culture via the induction of apoptosis. Further, these inhibitors provoked the separation of adult worm pairs in culture, a reduction in egg laying and Salermide treatment induced massive modifications to the ovaries and testes. Finally, the knockdown of *SmSirt1* transcripts by RNAi in adult worms led to similar modifications in the ovaries as seen after Salermide treatment.

## Materials and Methods

### Ethics statement

All animal experimentation was conducted in accordance with the European Convention for the Protection of Vertebrate Animals used for Experimental and other Scientific Purposes (ETS No 123, revised Appendix A) and was approved by the committee for ethics in animal experimentation of the Nord-Pas de Calais region (Authorization No. AF/2009) and the Pasteur Institute of Lille (Agreement No. A59-35009).

### Parasite material

A Puerto Rican strain of *S. mansoni* is maintained in the laboratory using the intermediate snail host *Biomphalaria glabrata* and the golden hamster *Mesocricetus auratus* as definitive host. Adult worms were obtained by whole-body perfusion of 6-week infected hamsters [31]. Eggs were obtained from the livers of infected hamsters and hatched out under light to obtain miracidia [32]. Newly transformed miracidia were maintained in complete Chernin's balanced salt solution [32] (CBSS) supplemented with 1 mg/mL glucose and 1 mg/mL trehalose, for 48 h to achieve *in vitro* transformation into primary sporocysts. Cercariae were released from infected snails, harvested on ice as described previously [33] and schistosomula were obtained *in vitro* by mechanical transformation [34]. Total RNA was isolated from the different stages of *S. mansoni* with TRIzol® reagent (Invitrogen) according to the manufacturer's instructions, followed by treatment with RNase-free DNase (Turbo DNA-free kit, Ambion).

### Molecular cloning of *S. mansoni* sirtuins

Sirtuins encoded in the *S. mansoni* genome were identified by screening using Hidden Markov Model profiles derived from the Pfam database [35]. Predicted protein sequences were manually annotated by integrating data from InterProScan [36] and reverse PSI-BLAST analysis. In order to verify and complete the predicted sequences, we carried out 5′ and 3′ RACE (GeneRacer Kit, Invitrogen) using oligonucleotides (Table S1) based on these sequences and generated full length cDNA sequences. The integrity of the sequences was verified by performing PCR using the Advantage 2 Polymerase mix according to the manufacturer's procedure (Clontech) and oligonucleotides encompassing the coding region. The PCR product was purified from agarose gels using the extraction kit Wizard SVGel and PCR clean up system (Promega) and inserted into pCR2.1-TOPO before transformation of chemically competent *Escherichia coli* cells (One Shot T0P10, Invitrogen). Selected clones were sequenced by GATC Biotech. Sequence analyses and alignments were performed using the LASERGENE package (DNAStar) and the BioEdit v7.1.11 package (http://www.mbio.ncsu.edu/BioEdit/bioedit.html).

### *In silico* analyses of *S. mansoni* sirtuins

Sirtuin conserved catalytic domain (Pfam: PF02146) sequences, delimited based on alignments in [15], were aligned using BioEdit v7.1.11 and the Clustal W program implemented therein. In order to determine the relationship of *S. mansoni* sirtuins to defined classes and families, we included sirtuin sequences from vertebrates, ecdysozoan invertebrates and one other lophotrochozoan (*Clonorchis sinensis*) in the analysis. An unrooted phylogram was generated using the MEGA4 neighbour-joining method [37] based on a Poisson correction substitution model and the figure was generated using FigTree (http://tree.bio.ed.ac.uk/software/figtree/). The confidence levels for the phylogenetic tree were estimated by bootstrapping using 100000 replicates. Secondary structures of sirtuins were obtained with a secondary structure prediction server, Jpred 3 [38] with human sirtuins as templates.

### Quantitative RT-PCR

Complementary DNAs were obtained by reverse transcription of total RNA using the Thermoscript RT-PCR System (Invitrogen) and used as templates in triplicate assays for PCR amplification using the SYBR Green PCR Master Mix and ABI PRISM 7000 sequence detection system (Applied Biosystems). Primers specific for *S. mansoni* sirtuins (Table S1) were designed by the Primer Express Program (Applied Biosystems) and used or amplication in triplicate assays. Measurements of real time PCR efficiency for each primer pair allowed the ratios of expression to be calculated using the 2^−ΔΔCt^ ratio [39] with *S. mansoni* α-tubulin as the reference transcript [40].

### Inhibitors

Sirtinol [41], MS3 (compound 26 in [42]), MS13 (compound 19 in [43]), HR103 (compound 5a in [44]), and CS13 (bis(4-nitrobenzylidene)pyrrolidine-2,4-dione; F.B., unpublished) were synthesized in our labs according to reported procedures. Purity and identity were assured using MS, NMR and HPLC. Salermide was purchased from Santa Cruz Biotechnology Inc.

### Toxicity of sirtuin inhibitors toward *S. mansoni* schistosomula and adult worms *in vitro*

The assay to determine the effects of sirtuin inhibitors on the viability of *S. mansoni* schistosomula was carried out as previously described [40]. Briefly, schistosomula (2000) were incubated at 37°C in a humid atmosphere containing 5% CO_2_ during 5 days in 6-well plates containing 2 mL of M199 medium (Invitrogen) kept at pH 7.4 with HEPES 10 mM and supplemented with penicillin (50 U/mL), streptomycin (50 µg/mL), gentamycin (15 µg/mL) and rifampicin (60 µg/mL) and 10% fetal calf serum (Gibco) (hereafter referred to as M199 complete medium) with two different concentrations (10 and 20 µM) of sirtuin inhibitors dissolved in DMSO. Culture medium was refreshed daily. Parasite mortality was assessed by eye each day using three criteria: absence of motility, tegument defects and granular appearance. A minimum of 300 larvae was observed for each condition, and the ratio of dead larvae to total larvae calculated. Two different assays were performed for each condition and three independent biological replicates (different batches of schistosomula) were carried out.

To measure the effect of sirtuin inhibitors on adult worm pairing in culture [45] *S. mansoni* adult worms obtained from hamsters were washed in M199 medium and ten pairs of adult worms were transferred to each well of a 6-well culture plate containing 2 mL of M199 completed medium. The worms were cultured during 6 days at 37°C in a humid atmosphere containing 5% CO_2_, and then two different concentrations of sirtuin inhibitors (10 and 20 µM) were tested. Culture medium and the inhibitors were refreshed daily. The number of paired couples was estimated every day by microscopy. In each well, medium containing the eggs was harvested every day and centrifuged. The total number of eggs was determined by microscopy and two different assays were performed for each condition and repeated with three independent biological replicates.

### TUNEL assay

Detection of DNA strand breaks in Salermide, Sirtinol and MS3 treated schistosomula was done using the Terminal deoxynucleotidyl transferase dUTP Nick End Labelling (TUNEL) method using the *In Situ* Cell Death Detection Kit, TMR red (Roche). The method designed for cell suspensions was followed as described in the manufacturer's instructions with modifications, Briefly, 2000 schistosomula were treated or not for 48 h with 10 and 20 µM Salermide, Sirtinol and MS3, in 6-well plates containing 2 mL of complete medium. Culture medium was removed and the schistosomula were centrifuged (1000 rpm, 2 min) washed three times in PBS, then fixed in formaldehyde 2% for 60 min. Schistosomula were washed once more in PBS and permeabilization solution (Triton X-100 0.1%, sodium citrate 0.1%) was added for 10 min on ice. Labeling of schistosomula with DAPI and TMR red dUTP was performed according to the manufacturer's instructions and TUNEL-positive parasites were observed by fluorescence using an AxioImager Z1-Apotome microscope (Zeiss).

### Confocal laser scanning microscopic examination

After 6 days in culture, worms were fixed for at least 24 h in AFA (ethanol 95%, formalin 3% and glacial acetic acid 2%), stained for 30 min with 2.5% hydrochloric carmine (Certistain, Merck), and destained in acidic 70% ethanol. Following dehydration in 70%, 90% and 100% ethanol, 5 min each, worms were preserved as whole-mounts in Canada balsam (Merck) on glass slides. To study the morphology of the reproductive organs of parasites, CLSM images were taken using a Leica TCS SP2 microscope with a 488 nm He/Ne laser and a 470 nm long-pass-filter under reflection mode.

### RNA interference in adult worms

Two fragments of 500 bp *SmSirt1*-dsRNA templates were generated by PCR using gene targeted primers containing T7 promoter sequences (Table S1). A luciferase dsRNA template of equivalent size was generated similarly using the pGL3-basic plasmid (Promega) as template. dsRNA was prepared and purified using the Megascript RNAi kit (Ambion) according to the manufacturer's instructions, and concentrations were determined spectrophotometrically (NanoVue Plus™, GE Healthcare). To deliver the dsRNA, 8 adult worms/group in 100 µL M199 medium containing 25 µg dsRNA, were electroporated in a 4 mm cuvette by applying a square wave with a single 20 ms impulse, at 125 V and at room temperature, as described [46]. Parasites were then transferred to 4 mL complete M199. After two days in culture, 2 mL of medium was removed and 2 mL of fresh complete M199 culture medium was added. Gene knockdown was monitored by qRT-PCR 5 days after dsRNA treatment as described above. Microscopic examination of RNAi-treated worms was carried out exactly as described below. Four independent experiments were carried out. The statistical significance of the level of *SmSirt1* transcript knockdown was evaluated using Student's t-test in the GraphPad Prism programme (GraphPad Software Inc.).

## Results

### Molecular cloning and characterization of *S. mansoni* sirtuins

Five protein sequences corresponding to sirtuins were identified in the *S. mansoni* predicted proteome [14] and initial sequence similarity searches using Blastp [47] provisionally identified them as potential homologs of mammalian sirtuins. The *S. mansoni* proteins include Sirt1 (Smp_138640), Sirt2 (Smp_084140), Sirt5 (Smp_055090), Sirt6 (Smp_134630) and Sirt7 (Smp_024670). Each of the corresponding coding sequences was present on a separate genome scaffold and each corresponded to a single copy gene. In order to verify the predicted sequences and to detect eventual splicing isoforms we carried out 5′ and 3′ RACE PCR using oligonucleotides based on the predicted coding sequences. This allowed us to confirm the existence of each of the predicted proteins as transcripts and to correct a number of assembly errors in the predicted sequences. The corrected sequences (including splice variants) have been submitted to the NCBI with accession numbers ABG78545 and KC993850 to KC993857.

**Figure 1:**
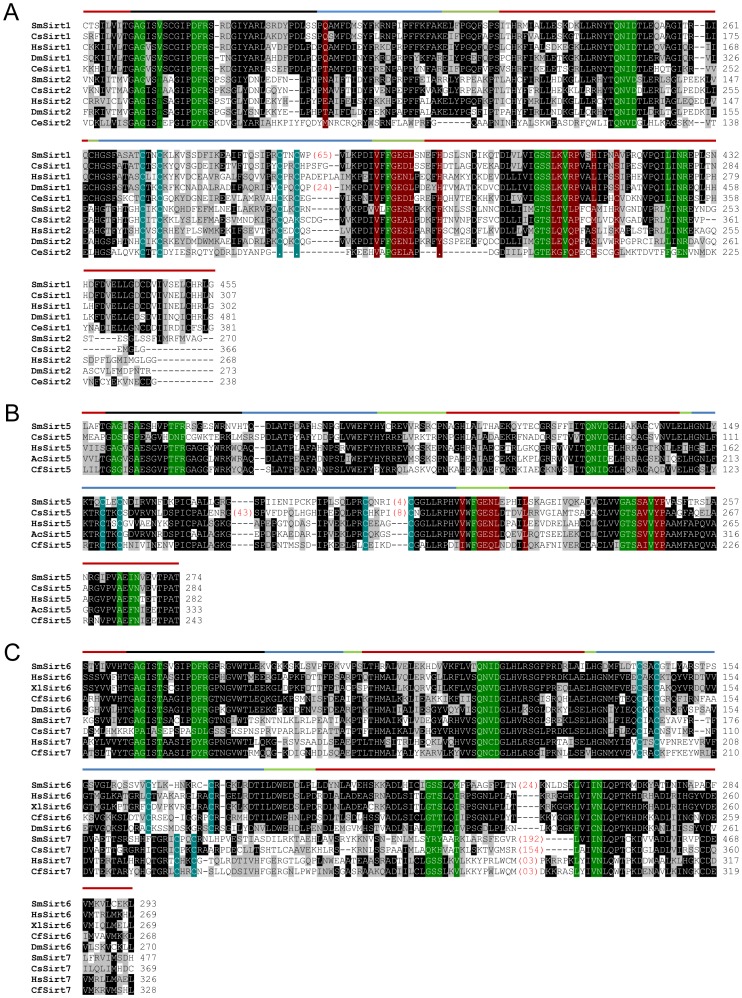
Alignment of the protein sequences of *S.* *mansoni* sirtuins catalytic domains with orthologues from other species. Sequences used in the alignment of sirtuin class I (Figure A) in addition to SmSirt1 and SmSirt2 are *Clonorchis sinensis* Sirt1 (CsSirt1 Accession No: GAA56043.1) Sirt2 (CsSirt2 GAA29763.1), *Homo sapiens* Sirt1 (HsSirt1: AAH12499.1) Sirt2 (HsSirt2: NP_036369.2), *Drosophila melanogaster* Sirt1 (DmSirt1: NP_477351.1) Sirt2 (DmSirt2: NP_650880.2), *Caenorhabditis elegans* Sirt1 (CeSirt1: NP_001255484.1) Sirt2 (CeSirt2: NP_001024673.1). Sequences used in the alignment of sirtuin class III (Figure B) in addition to SmSirt5 are *C. sinensis* Sirt5 (CsSirt5: GAA47482.1), *H. sapiens* Sirt5 (HsSirt5: NP_036373.1), *Aplysia californica* Sirt5 (AcSirt5: NP_001191422.1), *Camponotus floridanus* Sirt5 (CfSirt5: EFN62888.1). Sequences used in the alignment of sirtuin class IV (Figure C) in addition to SmSirt6 and SmSirt7 are *C. sinensis* Sirt7 (CsSirt7: GAA293.65.2), *H. sapiens* Sirt6 (HsSirt6: CAG33481.1) Sirt7 (HsSirt7: NP_057622.1), *Xenopus laevis* Sirt6 (XlSirt6: NP_001085592.1), *C. floridanus* Sirt6 (CfSirt6: EFN65179.1) Sirt7 (CfSirt7: EFN70308.1), *D. melanogaster* Sirt6 (DmSirt6: NP_649990.2) Sirt7 (NP_651664.2). Solid lines above the sequence alignment indicate which regions of the proteins compose the Rossmann-fold domain (red), cofactor binding loop (black), small domain (blue) and loop regions (green). Highlighted residues in the sequence alignment indicate which amino acids are involved in NAD+ binding (green), zinc binding (blue) and acetyl-lysine peptide binding (red).

The alignment of the catalytic domain (Pfam: PF02146) of *S. mansoni* sirtuins (SmSirt1, 2, 5, 6 and 7) with homologues from other species is shown in Fig. 1A, B and C. This alignment shows that although the schistosome sirtuin sequences diverge from those of vertebrates or ecdysozoan invertebrates, crucial residues involved in NAD+ binding, acetyl-lysine peptide binding or zinc binding are generally conserved, suggesting functional conservation of these sirtuins in the parasite.

The *SmSirt1* transcript (1832 nt) encodes a protein of 568 aa. The alignment of the catalytic domain of SmSirt1 (aa183–466) with orthologues from *C. sinensis*, *Caenorhabditis elegans*, *Drosophila melanogaster* and *Homo sapiens*, is shown in Fig. 1A. Although this domain is well conserved, showing an overall sequence identity of about 70% compared to all three sequences, it also has a large insertion of 63 aa, not shared with Sirt1 orthologues (Fig. S1). This insertion contains a putative PEST motif (KVDPSSLLPDEMNDSESTNH) at its N-terminal, characterized by an enrichment for proline, glutamate (or aspartate), serine and threonine, flanked by lysine, asparagine or histidine residues, that targets proteins for rapid destruction [48]. The short insertion present in *D. melanogaster* Sirt1 may also represent a PEST motif. The insertion in SmSirt1 further contains a C-terminal phosphorylation motif for PKB/Akt (Fig. S1) [49] that is also absent from all orthologues examined, including Sirt1 from the related trematode *C. sinensis* (oriental liver fluke). However, we were concerned that this insertion might represent either a cloning artefact, a splice variant or be specific for the Puerto-Rican strain of *S. mansoni* maintained in our laboratory. We therefore performed PCR, using primers flanking the insertion, on cDNAs from our strain and two isolates from Guadeloupe and Brazil (kind gifts from G. Mitta, UMR 5244, CNRS EPHE, University of Perpignan). Results (not shown) indicate that the same fragment is obtained whatever the *S. mansoni* strain tested, indicating that the only transcript produced contains the insertion. SmSirt2 is encoded by a unique transcript of 1460 nt that encodes a protein of 337 aa the sequence of which is identical to the prediction (Smp_084140).

In contrast, we identified five different *SmSirt5* transcripts, only one of which (isoform 4) seems to contain a complete catalytic domain. This isoform is encoded by a 1346 nt transcript (299 aa) and is the sequence shown in Fig. 1. However, even this sequence is not identical to the proteome prediction (Smp_055090), the latter starting at a Met residue 6 aa upstream of that of isoform 4, which was not confirmed by our RACE experiments. The discrepancy is due to the presence of an intron just upstream of the ATG start codon and a non-coding 5′ exon (not shown). The other isoforms detected are due to alternative splicing sites within exon 3, leading to the splicing out of parts of this exon, or to an alternative 3′ exon. These forms encode peptides with the catalytic domain truncated at the N- or C-terminal ends respectively (summarized in Fig. S2) the function of which remains to be determined.

Although the assembly and annotation of the *S. mansoni* genome has been improved since its initial publication [14], [50] some problems with gene assembly and annotation remain. The predicted protein sequence of SmSirt6 (Smp_134630) is a chimera between Sirt6 and a mannosyltransferase. Our RACE experiments allowed us to show not only that SmSirt6 is not chimeric, but also that about 80 aa were missing from the predicted sequence. Full length SmSirt 6 is a protein of 386 aa encoded by a single transcript of 1287 nt.

The coding sequence of *SmSirt7* was found to be identical to the proteome prediction, but close examination of the full-length transcript revealed that the first 33 nt at the 5′ end corresponded to the spliced leader sequence described by Davis et al [51] that is present on a subset of *S. mansoni* transcripts and that the translation initiating ATG codon was also provided by the spliced leader as described for other transcripts [52]. The full-length transcript (1782 nt) encodes a 517 aa protein, which, like SmSirt1, contains a large insertion (about 190 aa) within the catalytic domain (Fig. 1). This insertion is also conserved in the sequence of *C. sinensis* Sirt7 although in this species the insertion is slightly smaller.

### Molecular phylogeny of *S. mansoni* sirtuins

**Figure 2:**
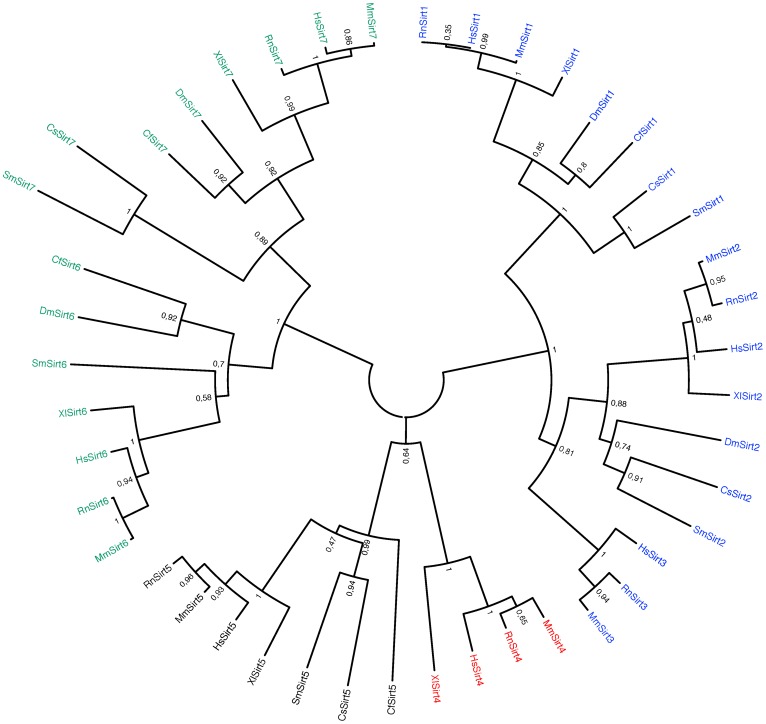
Phylogenetic analysis of *S.* *mansoni* sirtuins catalytic domains. The figure shows a phylogram generated by neighbour-joining analysis (see Methods) to emphasize the relationships between schistosome sirtuins and their orthologues. Class I sirtuins are shown in blue, Class II in red, Class III in black and Class IV in green. Sequences used were as in Figure 1 without *C. elegans* and *A. californica* sequences and with the addition of *H. sapiens* Sirt3 (HsSirt3: AAD40851.1) Sirt4 (HsSirt4: NP_036372.1), *Mus musculus* Sirt1 (MmSirt1: AAI52315.1) Sirt2 (MmSirt2: NP_071877.3) Sirt3 (MmSirt3: AAH25878.1) Sirt4 (MmSirt4: NP_598521.1) Sirt5 (MmSirt5: NP_849179.1) Sirt6 (MmSirt6: NP_853617.1) Sirt7 (MmSirt7: NP_694696.2), *Rattus norvegicus* Sirt1 (RnSirt1 XP_003753523.1) Sirt2 (RnSirt2 NP_001008369.1) Sirt3 (RnSirt3: NP_001099783.1) Sirt4 (RnSirt4: AAI67005.1) Sirt5 (RnSirt5 NP_001004256.1) Sirt6 (RnSirt6 NP_001026819.1) Sirt7 (NP_001100543.1), *X. laevis* Sirt1 (XlSirt1: NP_001091195.1) Sirt2 (XlSirt2: NP_001088636.1) Sirt4 (XlSirt4: NP_001084634.1) Sirt5 (XlSirt5: NP_001088966.1) Sirt7 (XlSirt7: NP_001088383.1), *C. floridanus* Sirt1 (CfSirt1: EFN72286.1).

In order to verify the assignment of orthologies of the *S. mansoni* sirtuins, we carried out phylogenetic analysis using neighbor-joining methodology. Other models were tested, but gave similar results. We present a phylogram (Fig. 2) that shows that all the *S. mansoni* sirtuins group with their orthologues within the four classes of eukaryotic sirtuins defined by Frye [15]. The figure omits the U class sirtuins present in archaea and bacteria. From this analysis it is clear that schistosomes have no orthologues of mammalian Sirt3 (classe Ib) or Sirt4 (class II). Along with Sirt5 the two latter sirtuins both localize to mitochondria in humans [20]. Consequently SmSirt5 is probably the only mitochondrial sirtuin present in *S. mansoni* and its predicted localization (PSORTII, [53]) is in agreement with this. Similarly, the predicted localizations of SmSirt1 (predominantly nuclear), SmSirt2 (cytoplasmic), SmSirt6 and SmSirt7 (nuclear) are the same as the effective localizations of their human orthologues.

### *S. mansoni* sirtuins are expressed at all life-cycle stages

**Figure 3:**
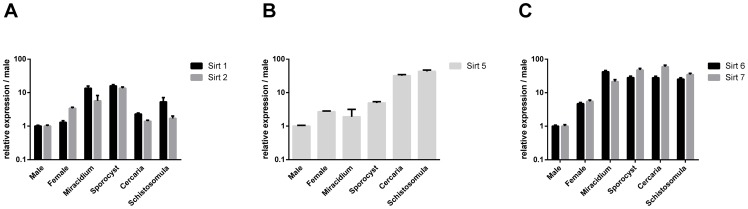
Quantitative of transcripts class I sirtuins (Figure 3 A), class III (Figure 3 B) and class IV (Figure 3C) at different *S.* *mansoni* life cycle stages. *SmSirt1*, *2*, *5*, *6* and *7* mRNA was measured in male and female adult worms, miracidia, sporocysts, cercariae and schistosomula by quantitative real-time PCR. *S. mansoni* α-tubulin was used as reference gene. Results are expressed as the log of 2^−ΔΔCt^ ratio compared to the expression in male worms taken arbitrarily as the base line.

Quantitative real-time RT-PCR was carried out at all parasite stages to determine the levels of expression of each of the *S. mansoni* sirtuin transcripts. In all cases the lowest level of transcript expression was detected in male adult worms and consequently transcript levels at other stages were expressed relative to this stage. Three distinct patterns of expression were evidenced, corresponding to the different sirtuin classes [15] (Fig. 3).The class I sirtuins, *SmSirt1* and *SmSirt2*, showed similar profiles of expression (Fig. 3A) with the highest transcript levels being present in miracidia and in sporocysts, whilst lower levels were present in male and female adult worms and in cercariae. However, maximal differences in expression levels throughout the life cycle were only about tenfold. *SmSirt5* mRNA (Fig. 3B), the only class III sirtuin identified, was most highly expressed in cercariae and schistosomula and much less (about 50-fold) abundantly in male adult worms. The expression profiles of *SmSirt6* and *SmSirt7* mRNAs (Fig. 3C), the class IV sirtuins, were very similar and showed the greatest amplitude of expression during the life-cycle. Indeed, both *SmSirt6* and *SmSirt7* transcripts were more than 50-fold more expressed in the larval stages than in male and female adult worms.

### Sirtuin inhibitors induce *in vitro* mortality and apoptosis in schistosomula

**Figure 4:**
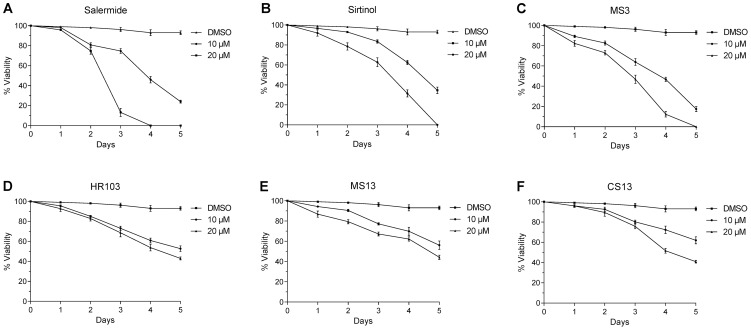
Viability of schistosomula maintained in culture up to 5 days in the presence or absence of 10 µM or 20 µM Salermide (Figure 4A), Sirtinol (Figure 4B), MS3 (Figure 4C), HR103 (Figure 4D), MS13 (Figure 4E) and CS13 (Figure 4F). Dead and dying larvae are dark and opaque and present granular aspect and tegumental damage. Results are expressed as mean % surviving larvae (± SEM, three independent experiments).

In order to determine whether *S. mansoni* sirtuins are potential chemotherapeutic targets, we chose a panel of six sirtuin inhibitors for testing on both schistosomula and adult worms in culture. Sirtinol is a potent Sirt1 inhibitor and treatment of MCF-7 human breast cancer cells with this inhibitor leads to hyperacetylation of the Sirt1/2 target p53 and induces apoptosis [25]. Salermide is an inhibitor of Sirt1 and Sirt2 [26] that causes apoptotic tumor-specific cell death in a variety of human cancer cell lines. MS3 and MS13 are thiobarbiturate inhibitors of Sirt1, Sirt2 [24] and the splitomicin derivative HR103 is a potent Sirt2 inhibitor. CS13 is a tetramic acid derivative. With the aim of determining the capacity of these sirtuin inhibitors to affect the viability of schistosome larvae maintained in culture, 3 h-old schistosomula were cultured for 5 days with a daily renewal of the medium containing the inhibitors. Parasite death was assessed by optical examination each day using three criteria: absence of motility, tegument defects and granular appearance. All sirtuin inhibitors tested induced the mortality of schistosomula in a time and dose-dependent manner (Fig. 4). However, Salermide (Fig. 4A), Sirtinol Fig. 4B) and MS3 (Fig. 4C) significantly reduced the viability of the schistosomula at 10 µM (respectively by 78%, 68% and 84%) and killed all the larvae at 20 µM after 5 days. These inhibitors were clearly more potent than HR103 (Fig. 4D), MS13 (Fig. 4E) and CS13 (Fig. 4F). For example, treatment at 10 and 20 µM with HR103, MS13 and CS13 affected parasite viability, but at a much lower level, with about 50% mortality after 5 days compared to the untreated controls incubated with DMSO alone.

**Figure 5:**
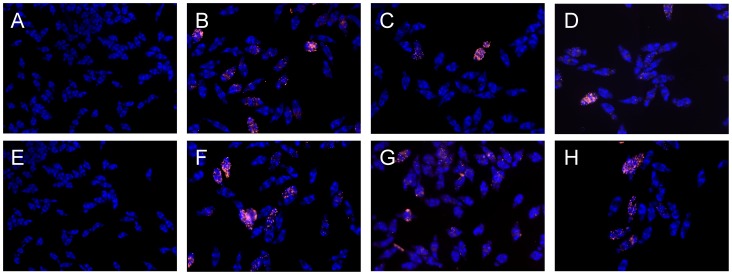
TUNEL labeling of parasite treated for 48 h with DMSO (A, E) or with 10 µM and 20 µM Salermide (B, F), Sirtinol (C,G) and MS3 (D,H) respectively. Positively TUNEL labeled schistosomula are red. The intensity of labeling, as well as the number of positively larvae, increased with the dose of sirtuin inhibitors used.

One of the principal effects of sirtuin inhibitors on cultured cancer cells is the induction of apoptotic cell death [25], [26]. Moreover, in our laboratory we have already shown that inhibitors of class I and II HDAC(s) induce apoptosis in larvae maintained in culture [40]. We therefore tested the capacity of sirtuin inhibitors to induce apoptosis in the cells of schistosomula using a TUNEL assay. In this experiment, schistosomula were treated with 10 or 20 µM of Salermide (Fig. 5B, 5F), Sirtinol (Fig. 5C, 5G) and MS3 (Fig. 5D, 5H) for 48 h, then fixed and stained with DAPI and TUNEL. The results indicate that these three inhibitors induce fragmentation of DNA, which may be due to the induction of apoptosis, within 48 h at the same concentrations that induced the mortality of the schistosomula within 3 to 5 days.

### Sirtuin inhibitors affect *in vitro* pairing and egg production of adult worms

**Figure 6:**
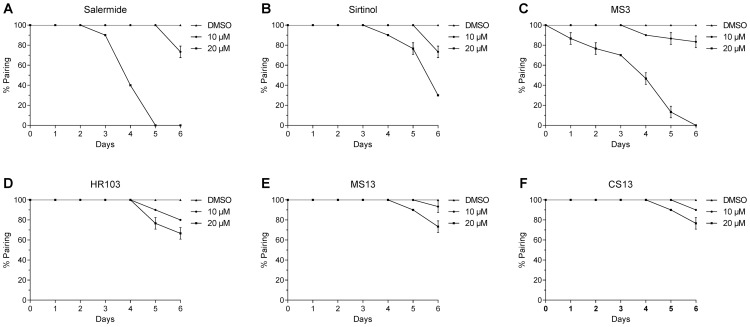
Freshly perfused adult couples were maintained in culture for 6 days and incubated with 10 µM or 20 µM of Salermide (A), Sirtinol (B), MS3 (C), HR103 (D), MS13 (E) and CS13 (F). DMSO solvent was use as the control. Percentages of paired worm were determined daily. The results are expressed as mean % paired worms (± SEM, three independent experiments).

To determine the effect of sirtuin inhibitors on adult worms, we decided to study the stability of pairing and the production of eggs of parasites maintained in culture. In these experiments, adult worm couples were treated with different concentrations of sirtuin inhibitors over a period of 6 days. The results indicate that during this period, sirtuin inhibitors affect pairing stability in a time and dose-dependent manner (Fig. 6). We showed that Salermide (Fig. 6A), Sirtinol (Figure 6B) and MS3 (Fig. 6C) had a greater effect on worm pairing than HR103 (Figure 6D), MS13 (Fig. 6E) and CS13 (Fig. 6F) compared to the controls incubated with the DMSO solvent alone. After 6 days of treatment with 10 µM of sirtuin inhibitors, worm couples were less affected than at 20 µM, with a reduction of 30% of the pairing for the most potent compounds (Salermide and Sirtinol) and a reduction of between 5% and 20% for the less potent compounds (MS3, HR103, CS13 and MS13). All worm couples had separated after 6 days of treatment with 20 µM of Salermide and MS3. Sirtinol at the same concentration induced a reduction of 70% in pairing.

**Figure 7:**
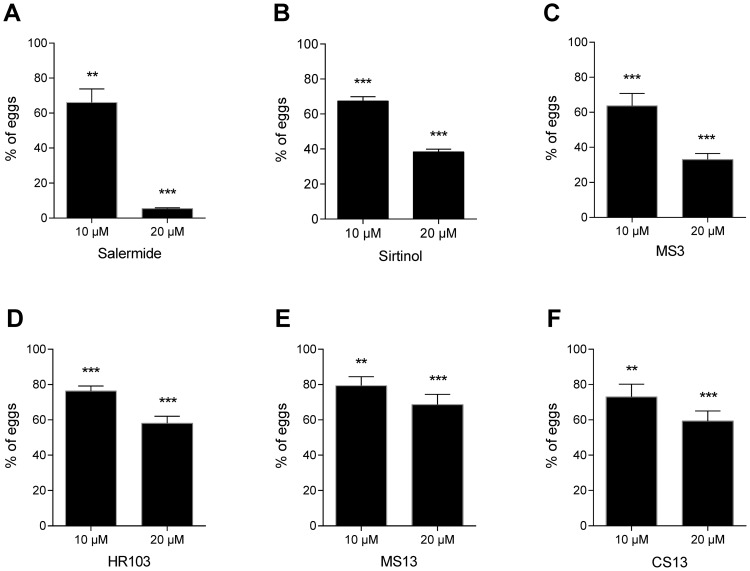
Total number of eggs laid during 5 days treatment with sirtuin inhibitors at 10 µM and 20 µM. Results obtained with (A) Salermide, (B) Sirtinol, (C) MS3, (D) HR103, (E) MS13 and (F) CS13 were expressed as mean % of eggs laid by treated worms compared to controls (± SD, three independent experiments). Statistical analyses were performed using student's t-test, and significance is displayed as follows: ** p<0.01, ***p<0.001.

We next determined the total number of eggs laid by the female worms during the 6 days of treatment for each sirtuin inhibitor tested. The results show that all sirtuin inhibitors decreased the number of eggs laid during the treatment compared to the control, in a dose-dependent manner (Fig. 7). At 10 µM, Salermide (Fig. 7A), Sirtinol (Fig. 7B) and MS3 (Fig. 7C) induced a reduction of about 30% in egg production and HR103 (Fig. 7D), MS13 (Fig. 7E) and CS13 (Fig. 7F) a reduction of around 20%. Treatment at 20 µM with Salermide had a drastic effect on egg production, inducing a decrease of 95% compare to the control. With Sirtinol and MS3, at the same concentration, we observed a lesser reduction around 60% and around 40% for HR103, MS13 and CS13.

**Figure 8:**
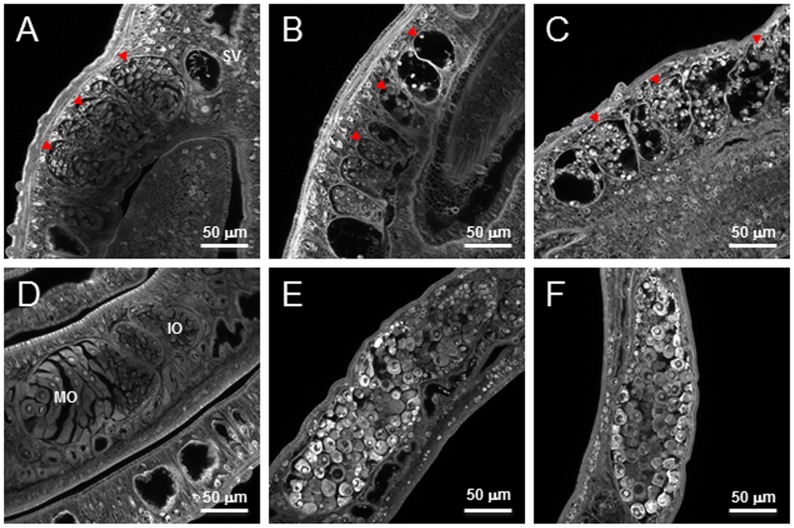
Freshly perfused adult couples were maintained in culture for 6 days and incubated with DMSO or with 10 µM and 20 µM of Salermide, then fixed and stained with hydrochloric carmine. Male worms treated with 10 µM (B) or 20 µM (C) Salermide show a drastic reduction in numbers of germinal cells in the testes (red arrows) compared to the control (A). The seminal vesicle is labeled (SV) on the control (A). In females treated with 10 µM (E) or 20 µM (F) Salermide we observed a dramatic disorganization of the ovary, where immature oocytes were less abundant and the mature cells seemed to invade the whole ovary compared to the control (D). The anterior part of the ovary, containing immature oocytes (IO) and the posterior part, containing mature oocytes (MO) are indicated.

To complement these results, we analyzed by confocal laser scanning microscopy the phenotypic effect on the ovary and the testis of adult worms treated with Salermide at 10 and 20 µM during 6 days. A remarkable effect on the morphology of the gonads of adult worms treated with this inhibitor was evidenced (Fig. 8). In untreated male worms, the testes are composed of several testicular lobes containing numerous spermatogonia and spermatocytes in different stages of maturation (Fig. 8A). However, after treatment with Salermide at 10 µM (Fig. 8B) we observed a drastic reduction in numbers of germinal cells in the testes and this effect was enhanced at 20 µM (Fig. 8C). In untreated female worms, the ovaries have an oval form and are composed of small oogonia with immature oocytes in the anterior part, and larger primary oocytes in the posterior part (Fig. 8D). In ovaries of worms treated with Salermide at 10 µM (Fig. 8E) we observed a dramatic disorganization, where immature oocytes were less abundant and the mature cells seemed to invade the whole ovary. As in the case of the male worm this effect was enhanced when the concentration of inhibitor was increased (Fig. 8F).

### Transcriptional knockdown of *SmSirt1* in adult worms leads to morphological changes in the ovary

**Figure 9:**
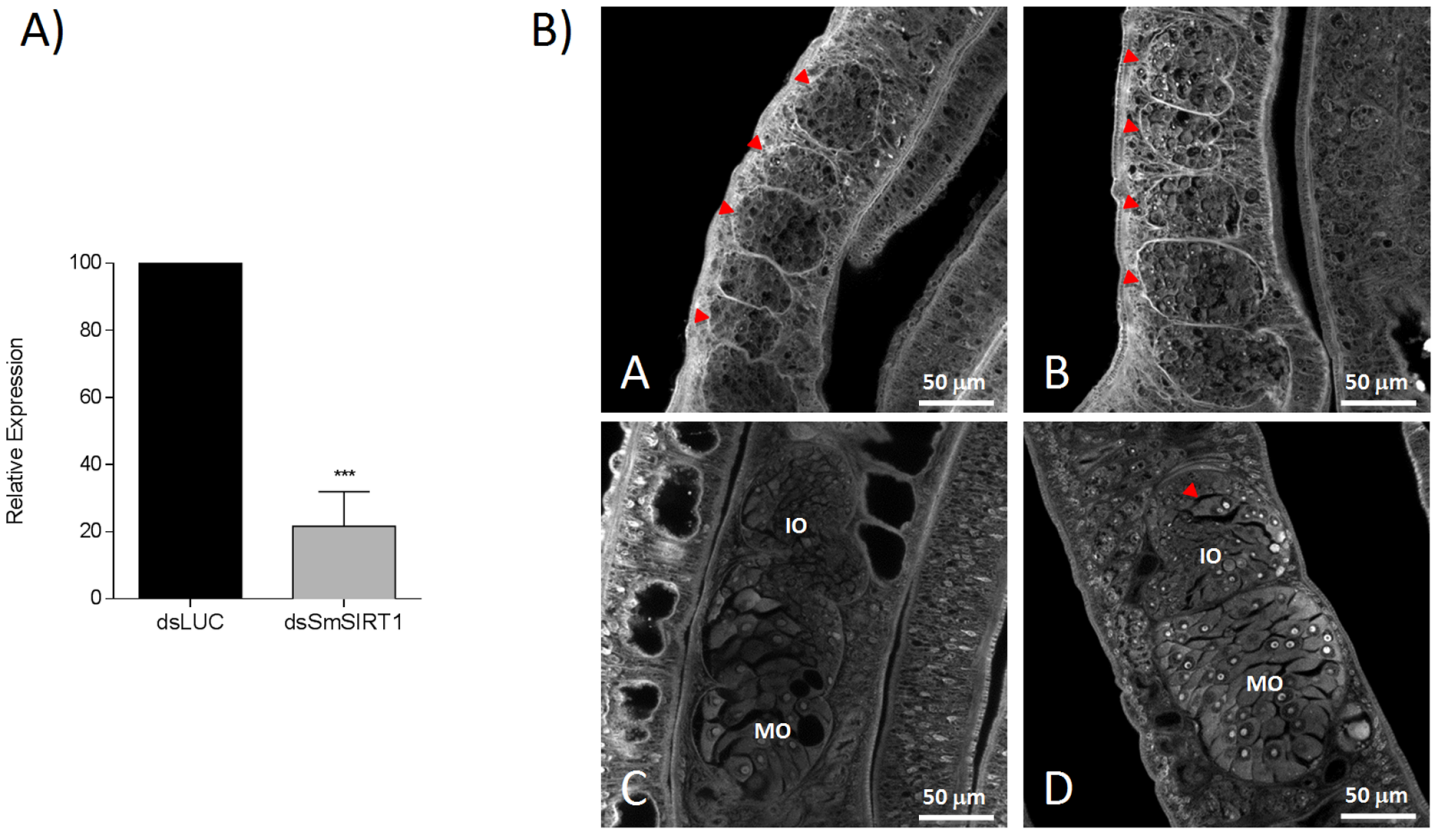
Effects of *SmSirt1* transcript knockdown in adult worms. RNA interference was carried out by the electroporation of adult worms with dsRNA for *SmSirt1* or *luciferase* (negative control) as described in the Methods section. (A) RNAi led to a reduction of about 80% in the level of transcripts for *SmSirt1* compared to controls knocked down for the irrelevant *luciferase* gene. (B) Microscopic examination was carried out 5 days after RNAi treatment as previously. This treatment caused no obvious morphological changes to male worm testes (B) compared to the control (A). Testes are indicated by red arrows. In contrast, RNAi treatment led to a marked increase in mature oocytes in the ovaries (D) compared to the control (C) particularly in the posterior part (MO). A mature oocyte in the anterior part of the ovary (IO), normally containing only immature oocytes (D) is arrowed. Results shown are representative of four independent experiments.

In order to determine whether Sirt1 inhibition was sufficient to explain the effects of the sirtuin inhibitors on adult worm morphology, we carried out RNA interference studies in adult worms to knockdown *SmSirt1* transcripts, followed by laser scanning microscopy of the male and female reproductive organs. The results (Fig. 9) show that *SmSirt1* knockdown leads to disorganization of the ovary, similar to that caused by Salermide treatment, with a marked increase in mature oocytes (Fig. 9B, D) and the appearance of mature oocytes in the anterior part of the ovary. However, no effects were seen on the testes (Fig. 9B, C), suggesting that the inhibition of other sirtuins by Salermide contributed to the phenotype observed after treatment with this drug. Four independent experiments were carried out with similar results.

## Discussion

The schistosome genome encodes orthologues of five of the seven sirtuins found in mammals. Since all seven are found in another lophotrochozoan, the oligochaete *Capitella capitata*, as well as in *Nematostella vectensis*, a radially symmetrical cnidarian, it is clear that representatives of all the sirtuin families were present in the common ancestor of metazoans, and in the ancestor of the lophotrochozoan lineage [19]. However, the absence of orthologues of Sirt3 and Sirt4 in *S. mansoni* and two other schistosome species, *S. japonicum* [54] and *S. haematobium* [55] indicates the loss of these genes from the schistosome lineage. Such losses of genes encoding one or other of the canonical sirtuin complement are frequent throughout metazoans. Among lophotrochozoans Sirt3 is absent from the leach *Helobdella robusta* and Sirt4 from the gasteropod *Lotia gigantea* [19]. Moreover, among the platyhelminthes, examination of the predicted proteins from the recently published *Echinococcus* genomes [56] suggests that both *E. granulosus* and *E. multilocularis* lack orthologues of Sirt4 and Sirt5, but possess an orthologue of Sirt3. In contrast, the trematode *C. sinensis* possesses representatives of all three mitochondrial sirtuins (3, 4 and 5) but apparently not Sirt6, which is localized in the nucleus.

The absence of genes encoding sirtuins with defined and vital functions could have three explanations: the corresponding genes were lost or inactivated (although the latter seems unlikely as no corresponding pseudogenes have been discovered), the genes have diverged at the sequence level from their homologs, preventing identification by standard methods, or that genomic sequence assembly errors prevent their identification. In view of the conservation of the protein sequences of sirtuins throughout the metazoa the second explanation seems unlikely. Moreover, the absence of any ESTs corresponding to the missing sirtuins suggests that gene assembly errors do not explain their absence either. This implies that other proteins in the genome have taken on the functions of the absent genes. The most parsimonious explanation is that the functions of Sirt3 and Sirt4 have been at least partially taken on by one or other of the remaining sirtuins. For instance, mammalian Sirt4 has recently been shown to regulate the metabolic response to DNA damage via the inhibition of the metabolism of glutamine [57]. The DNA damage response (DDR) is highly conserved and the components of the DDR network are present in almost all eukaryotes [58]. It therefore seems probable that in *S. mansoni*, which does not possess an orthologue of Sirt4, these functions have been taken on by the only mitochondrial sirtuin present, SmSirt5. Interestingly, this sirtuin was the only one for which transcripts encoding multiple splicing isoforms were detected. We can speculate that these isoforms fulfill different functions within the schistosome mitochondrion.

The expression of transcripts of all the *S. mansoni* sirtuins at all life-cycle stages, suggests that all perform important cellular functions. However, three distinct expression patterns were identified that correlated with the sirtuin classes. The class I sirtuins *SmSirt1* and *SmSirt2* have a relatively homogeneous level of transcript expression throughout the life cycle that may be in relation to their multiple functions within the cell. *SmSirt 5* (class III) on the other hand shows an approximate 50-fold increase in expression in cercariae and schistosomula compared to adult male worms. Since SmSirt5 is mitochondrial it is tempting to link this increase in expression with metabolic changes that occur during the transition from free-living cercariae to the tissue-dwelling schistosomula. Within 24 hours following cercarial transformation, schistosomula switch from generating energy from glucose predominately by oxidative phosphorylation to a dependence on lactate fermentation that is maintained in adult worms [9]. Since mitochondrial metabolism is controlled by sirtuins (notably Sirt3) in mammals (reviewed in [23]), we can speculate that the increased transcript level of *SmSirt5*, the only mitochondrial sirtuin present in the parasite, may be linked to this switch.

Sirt6, which is localized to the nucleus, is also a major player in the regulation of glucose metabolism [59] and is involved in the control of the transcription of genes encoding key enzymes in the glycolytic pathway by keeping histone H3K9 acetylation levels low in the corresponding gene promoters. In this way its activity favors the maintenance of oxidative phosphorylation, rather than lactate fermentation. In common with SmSirt7, which has recently been shown to specifically deacetylate H3K18 [60], *SmSirt6* transcripts are highly expressed (more than 50× the levels in adult male worms) in all the larval stages (miracidia, primary sporocysts, cercariae and schistosomula). This may tally with the maintenance of oxidative phosphorylation as the primary means of generation of ATP in the motile larvae (miracidia and cercariae) and in newly transformed sporocysts and schistosomula. However, in addition to its deacetylase activity, human Sirt6 also removes fatty acyl groups (myristoyl, palmitoyl) from lysine residues, notably from TNFα [18]. At the very least, this indicates that Sirt6 has functions other than the regulation of energy metabolism in mammalian cells, and by extension in schistosomes.

In our study we have determined the effects of selected sirtuin inhibitors on both schistosomula and adult worms maintained in culture. The compounds used have all been shown to inhibit human Sirt1, Sirt 2 and in some cases Sirt3 with IC_50_ values generally in the low µM range. Of these, Sirtinol, Salermide and MS3 were the most effective in inducing apoptosis and death in schistosomula and separation of adult worm pairs with a concomitant reduction in egg laying. Sirtinol is a hydroxynaphthaldehyde derivative and was shown to inhibit yeast Sir2p and Sirt2 [58], but in Hela cells treated with Sirtinol no evidence for the hyperacetylation of histones or tubulin was evidenced, although this has been contradicted in the case of tubulin in a later study [61]. Moreover, Sirtinol inhibits various enzyme classes *in vitro* by precipitation or aggregation [62]. Against this a recent study has shown that Sirtinol treatment did increase the acetylation level of the Sirt1 target p53 in MCF-7 breast cancer cells, as well as inducing cell death via both apoptotic and autophagic mechanisms [25].

Salermide is a Sirtinol analogue that has also been shown to selectively induce apoptosis in cancer cells [26]. Again this was independent of an effect on global tubulin or histone acetylation, but in this case was also independent of p53. The apoptotic effect was ascribed to the activation of proapoptotic genes repressed in cancer cells by Sirt1. However, an independent study [61] suggests that Salermide treatment does lead both to increased histone and tubulin acetylation and that apoptosis induction is p53 dependent.

The observed effects of Salermide on the testes and ovaries of adult worms suggest that *S. mansoni* sirtuins have crucial functions in schistosome reproduction. In particular, the reduction in the number of germinal cells in the testes may indicate that the role of sirtuins and in particular Sirt1 in spermatogenesis and germ cell function, which was demonstrated in Sirt1^−/−^ mice [63], is conserved in metazoan evolution. Moreover, Sirt1^−/−^ female mice are sterile, but this seems not to be linked to the absence of the Sirt1 enzyme activity since female mice carrying a point mutation of Sirt1, which abolishes its enzymatic activity whilst maintaining the expression of the Sirt1 protein [64], are fertile. Our observations following transcriptional knockdown of *SmSirt1* in adult worms suggest that this enzyme has a conserved role in female worm reproductive physiology, but not that of male worms. Whilst morphological changes induced by RNAi for *SmSirt1* in the ovary resembled those seen after Salermide treatment, no effect was seen on the numbers of germinal cells in the testes. The effect of Salermide on the latter may have been due to the inhibition of other sirtuins. However, our results indicate that sirtuin enzyme activity is necessary for both male and female worm reproductive functions and that SmSirt1 is a potential therapeutic target.

Salermide and Sirtinol inhibit Sirt1 and Sirt2 and they induce apoptosis in cancer cell lines, but not normal cell lines. MS3 is a thiobarbiturate inhibitor with three- to four-fold selectivity for Sirt1 over Sirt2 [24]. Our observation that all three of these inhibitors induce apoptosis and kill schistosomula, together with their effects on adult worms strengthens the credentials of *S. mansoni* sirtuins as drug targets. More generally, our results lend weight to the cancer/schistosome analogy and show that these parasites are particularly susceptible to drugs directed toward epigenetic targets.

### Accession numbers

The corrected sequences (including splice variants) of the *S. mansoni* sirtuins have been submitted to the NCBI with accession numbers ABG78545 (SmSirt1) KC993850 (SmSirt2), KC993851 (SmSirt5 isoform 1), KC993852 (SmSirt5 isoform 2), KC993853 (SmSirt5 isoform 3), KC993854 (SmSirt5 isoform 4), KC993855 (SmSirt5 isoform 5), KC993856 (SmSirt6) and KC993857 (SmSirt7).

## Supporting Information

Figure S1 SmSirt1 contains a large insertion within the catalytic domain. Alignment of the amino acid sequences of the catalytic domains of SmSirt1 and orthologues from *D. melanogaster* (DmSirt1: NP_477351.1), *X. laevis* (XlSirt1: NP_001091195.1) and *H. sapiens* (HsSirt1: AAH12499.1). A putative PEST motif and PKB phosphorylation site are overligned in red.
(TIF)

Figure S2 Alignment of the five SmSirt5 splicing isoforms. Alignment of the complete amino acid sequences of the isoforms shows the use of an alternative 5′ exon (isoforms 3 and 5), the use of alternative splicing sites within exon 3 (isoforms 1, 2 and 4) or the use of an alternative 3′ exon (isoforms 4 and 5).
(TIF)

Table S1 List of oligonucleotides used.
(XLSX)
