# Supplementary figures and images for: Schistosoma mansoni Sirtuins: Characterization and Potential as Chemotherapeutic Targets
Source: PLoS Negl Trop Dis. 2013 Sep 12;7(9):e2428. doi: 10.1371/journal.pntd.0002428 (PMC3772001; doi:10.1371/journal.pntd.0002428)

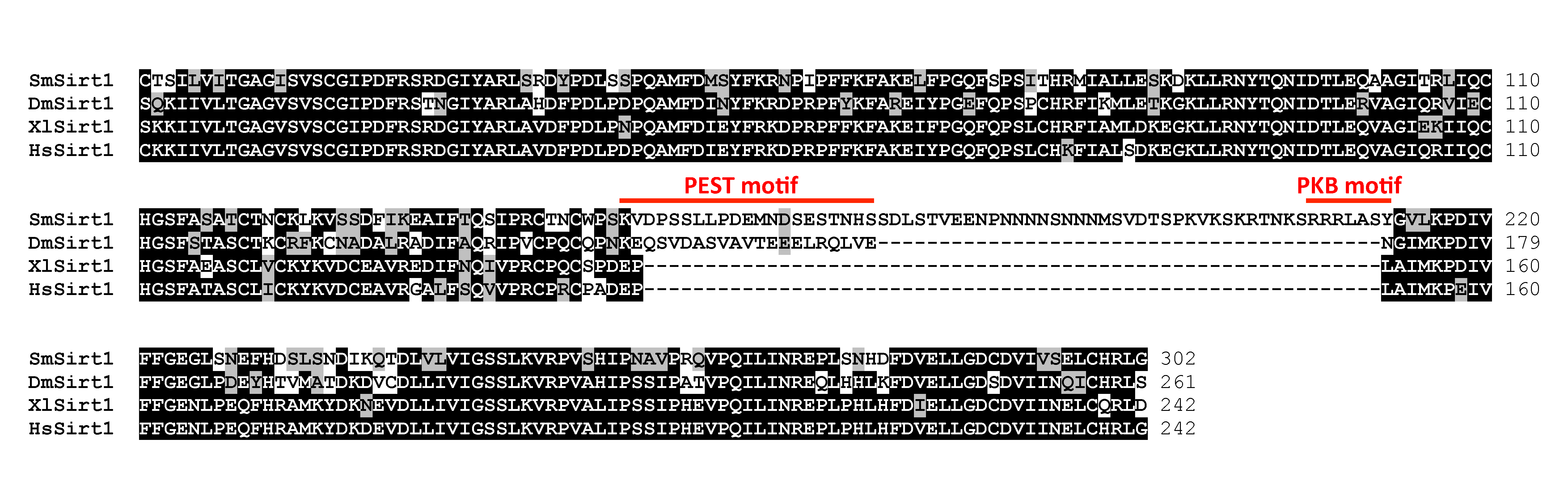

Supplement: Figure S1 — SmSirt1 contains a large insertion within the catalytic domain. Alignment of the amino acid sequences of the catalytic domains of SmSirt1 and orthologues from D. melanogaster (DmSirt1: NP_477351.1), X. laevis (XlSirt1: NP_001091195.1) and H. sapiens (HsSirt1: AAH12499.1). A putative PEST motif and PKB phosphorylation site are overligned in red. (TIF) [file pntd.0002428.s001.tif]

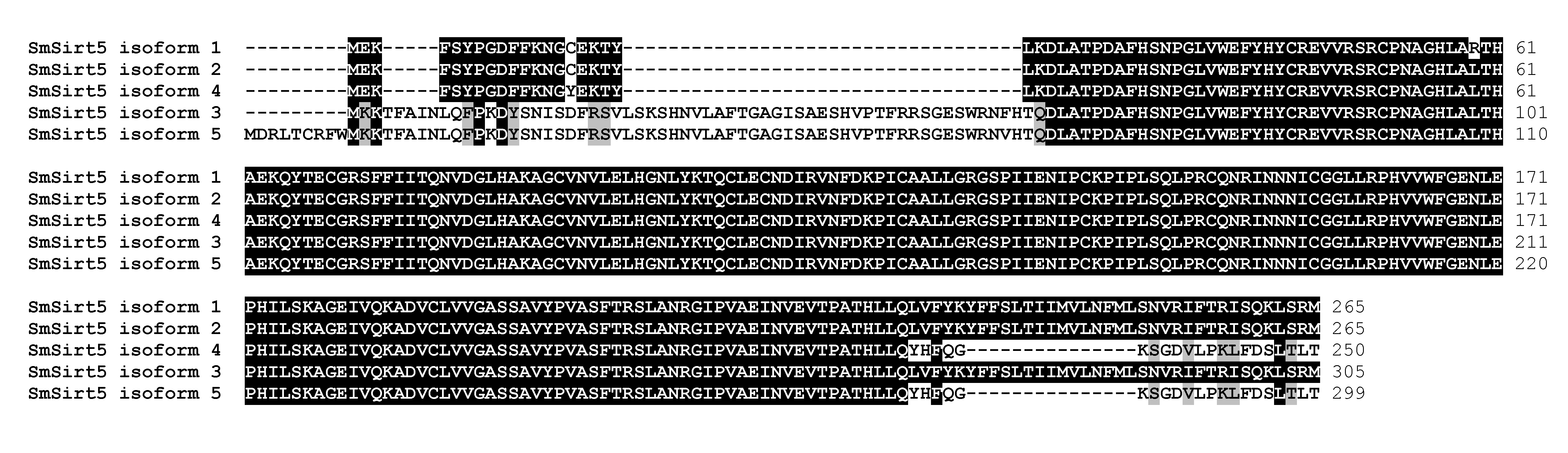

Supplement: Figure S2 — Alignment of the five SmSirt5 splicing isoforms. Alignment of the complete amino acid sequences of the isoforms shows the use of an alternative 5′ exon (isoforms 3 and 5), the use of alternative splicing sites within exon 3 (isoforms 1, 2 and 4) or the use of an alternative 3′ exon (isoforms 4 and 5). (TIF) [file pntd.0002428.s002.tif]
